# Unpasteurized milk from the bulk tank used for calf-feeding might increase incidence of BLV in dairy farms

**DOI:** 10.1186/1742-4690-12-S1-P38

**Published:** 2015-08-28

**Authors:** Natalia G Porta, Juan P Jaworski, Rozana Galarza, Geronimo Gutierrez, Romina P Politzki, Irene Álvarez, Luis Calvinhos, Karina G Trono

**Affiliations:** 1 Instituto de Virología, C.I.C.V. y A., Instituto Nacional de Tecnología AgropecuariaEEA Rafaela, Buenos Aires, Argentina

## 

We think that the ingestion of milk with provirus or free virus particles could be important for BLV vertical transmission in calves. In order to explore this idea we conducted an epidemiological study in an experimental milking facility (8,0 Kg/animal/year). The herd was composed by 332 dairy cattle and their calves were fed using non-pasteurized milk from the bulk tank located at the milking parlor. Blood and milk samples were obtained from 49 randomly selected animals. In addition, we collected 10 milk samples from the bulk tank along a one-month period. We tested all samples for the presence and quantity of BLV DNA and BLV-specific antibodies (Abs). We observed an individual serological prevalence of 94 % and 96 % considering the whole dairy herd (n=332) and the subgroup of animals (n=49), respectively. From the latest group we detected the presence of BLV DNA in 60 % and 30 % of blood and milk samples, respectively. In addition we detected BLV Abs in 90 % of individual milk samples. Then, we assayed milk samples from the bulk tank (n=10) detecting BLV Abs in all cases and the presence of BLV DNA in 8 out of 10 opportunities. Finally, we observed a weak positive correlation between the levels of cell associated viral load (CAVL) in blood and BLV Abs in serum and milk. The overall prevalence described here is in agreement with the one described for other farms with a similar level of production. Furthermore, we found that there were a higher proportion of milk samples carrying BLV DNA in the bulk tank compared to individual milk samples. Feeding calves using unpasteurized milk from this source could increase the risk of transmission by at least one of the following ways: (i) increasing the frequency of provirus intake by the calf and (ii) disrupting the natural balance between provirus and Abs that the calf receives directly from his mother.

